# A Deep Learning-Based Phenotypic Analysis of Rice Root Distribution from Field Images

**DOI:** 10.34133/2020/3194308

**Published:** 2020-10-16

**Authors:** S. Teramoto, Y. Uga

**Affiliations:** Institute of Crop Science, National Agriculture and Food Research Organization, 2-1-2 Kannondai, Tsukuba, Ibaraki 305-8518, Japan

## Abstract

Root distribution in the soil determines plants' nutrient and water uptake capacity. Therefore, root distribution is one of the most important factors in crop production. The trench profile method is used to observe the root distribution underground by making a rectangular hole close to the crop, providing informative images of the root distribution compared to other root phenotyping methods. However, much effort is required to segment the root area for quantification. In this study, we present a promising approach employing a convolutional neural network for root segmentation in trench profile images. We defined two parameters, Depth50 and Width50, representing the vertical and horizontal centroid of root distribution, respectively. Quantified parameters for root distribution in rice (*Oryza sativa* L.) predicted by the trained model were highly correlated with parameters calculated by manual tracing. These results indicated that this approach is useful for rapid quantification of the root distribution from the trench profile images. Using the trained model, we quantified the root distribution parameters among 60 rice accessions, revealing the phenotypic diversity of root distributions. We conclude that employing the trench profile method and a convolutional neural network is reliable for root phenotyping and it will furthermore facilitate the study of crop roots in the field.

## 1. Introduction

Root distribution in the soil is a major component of root system architecture [1] that affects crop growth and yield [2] because nutrients and water distribution in the soil are uneven. In cultivated fields, nutrient distribution varies depending on the area of cultivation, tilling method, and fertilizing system. Therefore, the root distribution is influenced by tillage and fertilizer placement [3, 4]. Generally, in modern cultivation using high-input systems, soils in plow layers, which are located near the ground surface, become highly fertile. Therefore, shallow-rooting crops are capable of capturing the nutrients and resulting in favorable growth [5]. On the contrary, in nutrient and water-deficient fields, such as those that occur during drought conditions, deep-rooting crops perform better by avoiding nutrient and water deficits [5, 6]. Together, this indicates that the root distribution is affected by the environmental conditions and cultivation techniques. Yield could be improved by breeding varieties with the root distribution suiting for the target farm [7, 8]. The success of breeding depends on how useful genetic resources can be found among natural and artificial populations using effective phenotyping methods. However, breeding new cultivars for an ideal root distribution is difficult, because phenotyping methods for root distribution in the field—a step which is required for screening genetic resources that modulate root distribution properties—remain limited and technically challenging [9].

The all available methods for root phenotyping are roughly classified into four groups [9]: trench profile, auger, minirhizotron, and direct excavation. The trench profile method is a technique to observe the vertical and horizontal root distribution of crops [10] by digging a vertical ditch beside the plant to quantify the distribution of roots present in the profile wall. The proportion of observable roots is the largest compared to the other field methods, and this technique is used for studying soil condition–root growth interactions [11, 12] and for the basic characterization of crop root distribution [13]. The auger method is a sampling method primarily used to quantify vertical root distribution [10]. Using a core sampler, the auger method can evaluate the root distribution only in narrow horizontal planes [14, 15]. The minirhizotron method, which has a limited observable area, is a technique to sequentially observe root development by burying a transparent cylinder to periodically acquire root images [16]. This technique is used for estimating root growth dynamics including root turnover [17, 18]. The direct excavation method is a simple root sampling method in which the roots are dug up using shovels [19]. Hence, it is not applicable for studying root distribution in the soil and is mainly used for root branching estimation and root cone angle measurement [19, 20]. Although these methods cannot observe the entire root distribution at once, the trench profile method is the most suitable for evaluating root distribution in the soil [21] because both the vertical and horizontal root distributions can be observed.

The trench profile method procedure consists of excavating the trench, flushing the trench profile wall, measuring the root length, and calculating the root distribution properties. Typically, the trench is dug perpendicularly using a backhoe [11, 22]. The distance between the trench and plant influences the root distribution on the profile wall [12, 23]. Furthermore, the depth of the trench should be determined by the rooting characteristics of the target crops [12, 13, 22]. Root distribution on the profile wall is evaluated by sectioning the wall vertically and horizontally and measuring root density in each section. For measuring root density, soil blocks [12] or core samples [22] were collected from each section, and root length was then measured. Otherwise, after removing a few centimeters of soil from the wall surface using a scraper, air pressure, or nebulizer for easy root observation and measurement [10], the root density in each block was estimated by root intersection counting based on their correlation relationship [23]. Thus, the trench profile method is laborious. Root segmentation for the trench profile images is a particularly time-consuming process.

In recent years, convolutional neural networks (CNN) have dominated image analysis for plant phenotyping [24, 25]. CNN is a deep learning model that is generally composed of four types of layers—convolution, pooling, de-convolution, and fully connected layers. The convolution and pooling layers extract features of the image, and output data are created by the deconvolution and fully connected layers. If the output data is an image, it is used for semantic segmentation and object detection tasks—examples of this application are a vein morphological patterning study [26], heading date estimation [27], and plant disease detection [28]. If the output data is a vector, it is used for classification tasks, such as plant species classification [29]. In case of cultivated plants, measuring plant roots is usually done using CNN, by a root box system [30] and minirhizotron [31, 32]. Root length was quantitatively calculated from semantic segmentation of the root system [30–32]. A CNN-based application becomes fully automatic once the model is trained, and hence, it could be a powerful tool for image-based plant phenotyping in the field. However, there are few CNN-based applications that can measure root distribution in the soil on the field.

In this study, we applied CNN-based semantic segmentation to the trench profile images for estimating root distribution parameters on the profile wall. The trench profile images were taken in the field, semantically segmented, and the root segments were skeletonized to represent root length. Maximum root depth (MRD) and root depth index (RDI) have both been widely used as parameters for root distribution [33–38]. MRD is the maximum depth root reached, and RDI is the vertical centroid of root distribution in the soil. Because only roots on a profile wall are visible with the trench profile method, there is a risk of not maximizing the MRD. Therefore, RDI is more suitable for the trench profile method. We have extended the idea of RDI to vertical and horizontal distributions; Depth50 and Width50 represent the vertical and horizontal centroids of root distribution, respectively. We showed its usefulness by evaluating the diversity of root distribution among worldwide rice accessions. The processes presented in this study could be adapted for root distribution measurements in other crops, not only in rice.

## 2. Materials and Methods

**Figure 1:**
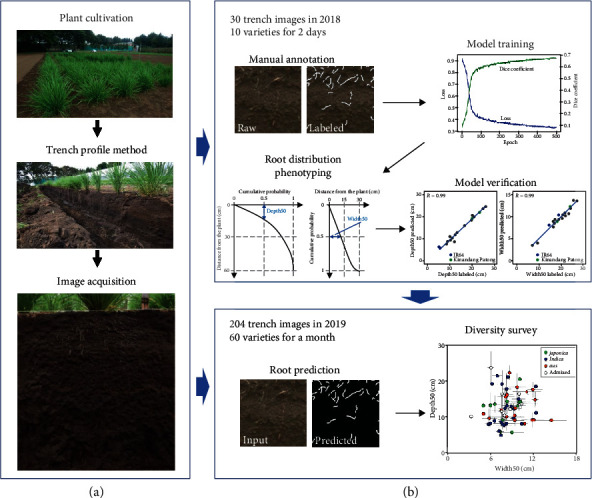
Overview of root distribution parameter estimation. (a) Rice plants were cultivated in the field; the ditch was created with a backhoe, and the trench profile image was acquired. (b) Upper right: in 2018, 30 trench profile images were manually annotated and used for model construction. Two root distribution parameters were defined, and the trained model was validated. (b) Lower right: in 2019, 204 trench profile images were used to assess the phenotypic diversity of root distribution among worldwide rice accessions.

An overview of this study is shown in Figure 1. In 2018, we obtained 30 trench profile images from ten rice accessions, which were used for model training and model validation to predict the root distribution in the soil. In 2019, we obtained 204 trench profile images from 60 accessions. These images were used for the evaluation of root distribution diversity among worldwide rice accessions. The methods are detailed in the subsections below.

### 2.1. Plant Cultivation

Sixty-one lines of rice comprising of 57 rice accessions from “NIAS Global Rice Core Collection” [39], Koshihikari, IR64, Kinandang Patong (KP), and Dro1-NIL were used in this study. Detailed information of the 61 accessions is shown in Table S1; subspecies were assigned according to a previous study [40]. Among the 61 accessions, 10 were used in 2018, and 60 were used in 2019. Koshihikari is a Japanese temperate *japonica*, one of the most popular cultivars in Japan [41]. IR64 and KP are *indica* and tropical *japonica* cultivars, respectively. Dro1-NIL is an IR64 near-isogenic line harboring the deep-rooting allele of the *DEEPER ROOTING 1* gene derived from KP [7]. In this study, we used IR64, KP, and Dro1-NIL as representative varieties; the average root diameter of KP is thicker than that of IR64 and Dro1-NIL, and the rooting angle of KP is the deepest, followed by those of Dro1-NIL and IR64 [42].

The field experiment was conducted in 2018 and 2019 at an upland field of the Institute of Crop Science (National Agriculture and Food Research Organization, Ibaraki, Japan; 36°02′89^″^ N and 140°09′97^″^ E), on volcanic ash soil of the Kanto loam type (Humic Andosol), at the same location as our previous study [42]. The topsoil (0–30 cm) is a dark humic silty loam, and the subsoil (below 30 cm) is a red-brown silty clay loam. There is a hardpan at a depth of approximately 20–25 cm. Fertilizer of 5.2 g N m^–2^, 15.4 g P_2_O_5_ m^–2^, and 5.6 g K_2_O m^–2^ was supplied before rice planting. In 2018, 10 plots were designed for 10 accessions (Table S1, Figure S1). Each plot consisted of 20 (5 × 4) hills. In 2019, 68 plots were designed for 60 accessions (Table S1, Figure S2). Each plot consisted of six (3 × 2) hills. In both 2018 and 2019, the hill spacing was 1m × 1m and three seeds were sown in each hill on June 5. On July 4, 2018, and on July 3, 2019, two seedlings were removed from each hill. If the remaining one seedling showed growth defects, it was replaced with an intermediate-growth seedling from another hill. Three hills in each plot were used for the trench profile method. To avoid severe drought stresses, water was supplied with a sprinkler before the start of leaf rolling.

### 2.2. Trench Profile Method and Image Acquisition

The trench profile images were obtained on August 29 and 30 in 2018 and from August 28 to September 26 in 2019, in the order the rice heading date (https://www.gene.affrc.go.jp/index_en.php). All trench images were acquired at or just before rice heading. To excavate the ditch, a B27 backhoe (YANMAR Co., Ltd., Osaka, Japan) was used in 2018 and a U-40-6E backhoe (KUBOTA Co., Osaka, Japan) was used in 2019. A ditch approximately 100 cm deep and 150 cm wide was dug approximately 5 cm in horizontal distance from the rice plant. About 1–2 cm of the soil layer on the profile wall was flushed with water using an agricultural nebulizer. The exposed roots on the profile wall were imaged with digital cameras: a D70 (NICON Co., Tokyo, Japan) and a D5600 (NICON Co., Tokyo, Japan) were used in 2018 and 2019, respectively. The image was taken in the trenches, horizontally from a distance of approximately 1 m from the wall and 30 cm below the ground surface. Each image included a scale bar, and the area of the profile wall had a depth of at least 60 cm and a width of at least 60 cm. Three images from three individuals in each plot were obtained. Lens distortion was corrected using GIMP software, version 2.8.22 (https://www.gimp.org/). All images were normalized by rotation, scale changes, and trimming at 60 cm depth and 60 cm width, with 256 dots per 10 cm.

### 2.3. Manual Annotation

All roots in the trench profile images were manually labeled with GIMP software. Each image was overlaid with a transparent layer, and the roots in the image were labeled using a pencil tool of 4-pixels width (approximately 1.56 mm) width, regardless of the root width. The labeled layer was converted into a black and white image and exported as an 8-bit image file.

### 2.4. Model Training

A U-shaped fully convolutional network was used to semantically segment the roots in the trench profile images. We used U-Net network architecture [43] with some modifications (Figure S3). It contains the contracting path and the expansive path, which have connection paths wired between them, resulting in a U-shaped architecture. The following three steps constitute the significant modifications from the original U-Net architecture: (1) in the convolution step, zero padding was accompanied to keep the image size. (2) Batch normalization steps were implemented. The up and down sampling steps were followed by batch normalization. (3) The output image was a single channel image because the number of classes in this study is one. Segmentation performance was gauged with the Dice coefficient, which is defined in Equation (1), where *T* is the manual segmentation and *P* is the predicted segmentation:

(1) $$\begin{array}{c} \text{Dice coefficient}=\frac{2\left|T\cap P\right|}{\left|T\right|+\left|P\right|}. \end{array}$$

The normalized trench profile images and the manually labeled images were preprocessed before the model training (Figure S4). Each image was divided into 36 tiles (6 × 6). The size of each tile was 10 × 10 cm or 256 × 256 pixels. A set of two tiles of the trench profile and labeled images was used as a training image set. They were subjected to a data augmentation step to increase the robustness of the model; image shape, such as angle and scale, and image color, such as intensity, gamma index, and chroma, were adjusted. The parameters of data augmentation are listed in Table S2, and representative results of data augmentation are shown in Figure S5. The model was trained using the Adam optimizer, of which learning rate was 0.001, for 500 epochs (360 inputs per an epoch) using 360 training image sets from 2018. The model trained for 500 epochs was used in this study.

### 2.5. Root Segmentation of Trench Profile Images

The trench profile image was divided into 36 tiles, as described in Model Training. Root segments of the 36 tiles were predicted by the trained model and assembled in the original order.

### 2.6. Root Distribution Phenotyping

**Figure 2:**
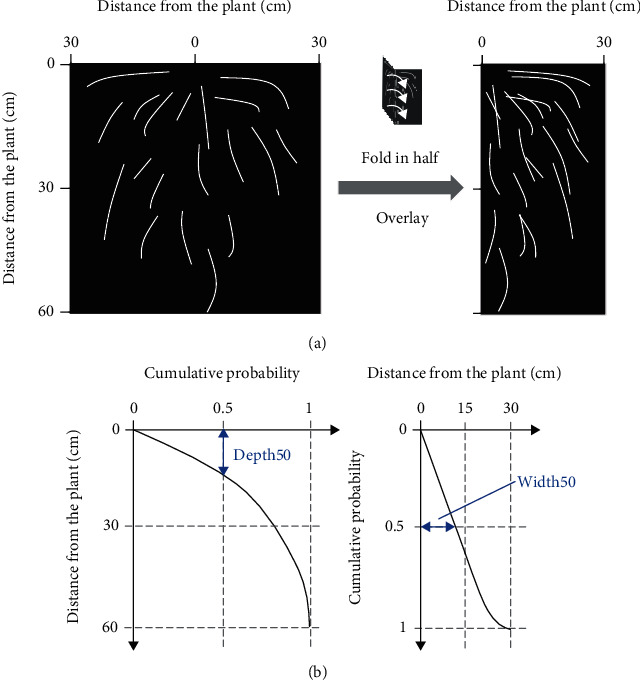
Calculating root distribution parameters. (a) The trench profile image was normalized as 60 cm in depth by 60 cm in width, with 256 dots per 10 cm. The image was folded in half and skeletonized to calculate the root distribution parameters. (b) A schematic diagram of how to calculate the root distribution parameters. In the case of Depth50, the cumulative probability along the vertical axis was calculated; the depth, with its cumulative probability of 0.5, was defined as Depth50. In the case of Width50, the cumulative probability along the horizontal axis was calculated; the width, with its cumulative probability of 0.5, was defined as Width50.

Root distribution parameters were calculated using Python3 (https://www.python.org/). The manually labeled or predicted image sized 60cm × 60cm was loaded as a NumPy array [44] and was horizontally folded in half (Figure 2(a)). To obtain the root length, the image was skeletonized by an image-processing module, scikit-image [45]. Because the resolution of the image was 25.6 dots per centimeter, one skeletonized pixel represents approximately 0.39 mm of root length. Centroid was used for phenotypic analysis of plant root distribution; we defined two root distribution parameters related to the centroid—Depth50 and Width50. Depth50 is the vertical distance from the surface of the soil, and the area within Depth50 includes 50% of the total root length. Width50 is the horizontal distance from the hill, and the area within Width50 includes 50% of the total root length. Depth50 and Width50 are annotated in Figure 2(b). Broad-sense heritability (*H_2_*) of Depth50 and Width50 was calculated with Equation (2), where *V*_*G*_ is the total genetic variation and *V*_*P*_ is the phenotypic variation:

(2) $$\begin{array}{c} H_{2}=\frac{V_{G}}{V_{P}}. \end{array}$$

### 2.7. Model Validation

The trained model was validated by comparing Depth50 and Width50 between manually labeled images and predicted ones. Twenty trench images from 2018, which were not used for model training, were used for validation. The Pearson correlation coefficient was calculated by the ‘cor' function in R version 3.5.1 (https://www.r-project.org/).

### 2.8. Diversity Survey

The diversity of the root distribution among worldwide rice accessions was evaluated. Of the 204 trench profile images taken in 2019, 27 images were of KP derived from nine plots and taken from August 28 to September 26. They were used for the evaluation of the influence of the acquisition date on root distribution. Depth50 and Width50 of the 60 accessions were calculated with at least three trench profile images. Statistical subspecies comparison was performed with a Steel–Dwass test due to the lack of normality in the distribution of the global rice collection; a *p* value typically < 0.05 was considered statistically significant. For the clustering analysis, heatmap and dendrogram were produced using “heatmaply” program [46]. Depth50 and Width50 were normalized with mean 0 and variance 1. A neighbor method was then used as an algorithm in the clustering analysis.

## 3. Results

### 3.1. Construction of the Prediction Model

**Figure 3:**
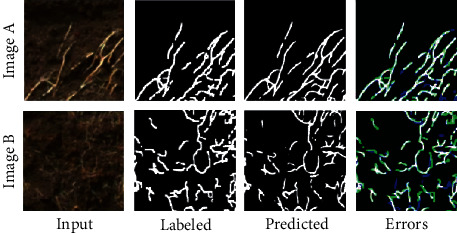
Representative results of local root segmentation. Two predicted tile images (Images A and B) are shown. Input: raw images; Labeled: manually labeled images; Predicted: predicted images by the trained model; Errors: differences between the manually labeled and predicted images. White indicates shared segments between the manually labeled and predicted images. Green and blue indicate segments which appear only in manually labeled images or only in predicted images, respectively.

We trained the U-shaped fully convolutional network using 10 trench profile images from 2018 and the 10 corresponding manually labeled images. The datasets that were used are shown in Figure S6, including high-contrast images arising due to strong sunlight and images containing different soil colors. After training for 500 epochs, the Dice coefficient of the prediction model was reached at approximately 0.67. Representative results of the prediction model are shown in Figure 3. The overall distribution of the roots in the manually labeled and predicted images is similar, but there are local differences between them. One source of the local differences could be that the predicted roots were thinner than the labeled roots. The labeled roots were traced using a pencil tool with a width of 4 pixels, regardless of the root thickness. Since the actual thickness of roots varies, the Dice coefficient seems to decrease.

**Figure 4:**
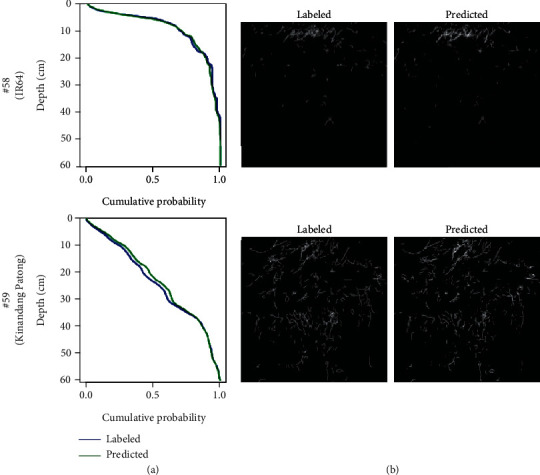
A representative result of entire root segmentation. Two results, IR64 and Kinandang Patong, are shown. (a) The cumulative probability-depth graph. The lines of manually labeled and predicted images are shown. The numbers displayed at the left are the IDs in Table S1. (b) Entire root segments analyzed by manual labeling (Labeled) and by trained model prediction (Predicted).

**Figure 5:**
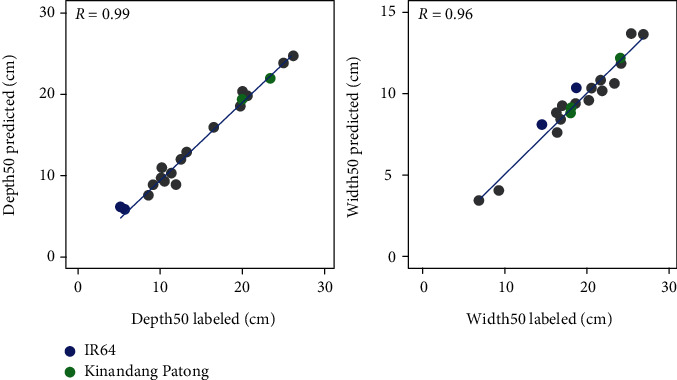
Scatter plots of Depth50 and Width50 in 10 rice accessions. The results of manually labeled and predicted images were compared. *R*: Pearson correlation coefficient.

The prediction model was validated using 20 trench profile images from 2018 and their corresponding 20 manually labeled images; manually labeled and predicted images were then compared. Depth-cumulative probability curves, manually labeled images, and predicted images of IR64 and KP are shown in Figure 4, and those of the other eight varieties are shown in Figure S7. Depth-cumulative probability curves show high similarity between manually labeled and predicted images in two varieties (Figure 4(a)), although the local root distribution between manually labeled and predicted images was different (Figure 4(b)). We calculated Depth50 and Width50 of both manually labeled and predicted images in 10 rice accessions and found a high correlation between the manually labeled and predicted images, where *R* = 0.99 and 0.96, respectively (Figure 5). The difference of depth-cumulative probability curves of IR64 and KP (Figure 4(a)) and the lower Depth50 value of IR64 compared to KP (Figure 5) supported that KP more proliferated their roots in a deeper soil region than IR64 did as previously reported [7]. Taken together, these results indicate that qualitative characteristics of root distribution can be estimated by deep learning-based phenotypic analysis.

### 3.2. Application of CNN Root Characterization Technique on Worldwide Rice Accessions

The trench profile method is time-consuming. It required two days to acquire 30 trench profile images: one day for making the ditch and one day for image acquisition and reclaiming the ditch. In 2019, we acquired 204 trench profile images in a month. Hence, there is a possibility that the images acquired on the first and last days show different root distribution parameters. To evaluate the influence of acquisition date on Depth50 and Width50, we obtained 27 trench profile images consisting of a single variety, KP, taken on seven days from August 28 to September 26. Depth50 and Width50 were calculated from the 27 trench profile images (Figure S8). The analysis of variance (ANOVA) indicated that there are no significant differences between acquisition dates in both Depth50 and Width50 (*α* = 0.05). This result suggested that the root distribution parameters were not affected by the acquisition date during the late growth stage in rice.

**Figure 6:**
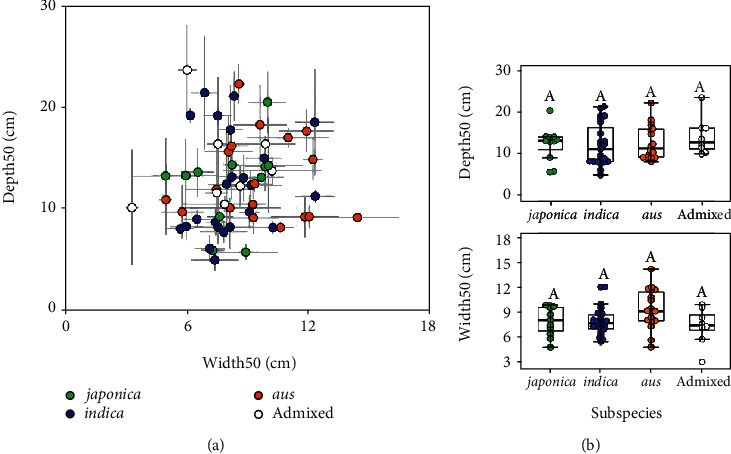
Diversity of Depth50 and Width50 among worldwide rice accessions from 2019. (a) Scatter plot of worldwide rice accessions cultivated. Sixty rice accessions were divided into 4 subspecies, namely, *japonica*, *indica*, *aus*, and admixed. (b) Box plot of Depth50 and Width50 among subspecies. The constituents of the box plot are marked as bee swarm points. The letters above the plots indicate a significant difference calculated by the Steel–Dwass test.

The 204 trench profile images in 2019 contained 60 accessions including 57 ones of a worldwide rice collection (Table S1). We tested whether the deep learning-based phenotypic approach is applicable for evaluating the diversity of root distribution parameters, Depth50 and Width50 in the case of this study, among worldwide rice accessions. Among 204 trench profile images, we obtained 201 predicted images. The remaining three were not predicted properly, possibly due to a very strong contrast caused by the setting sun. Thus, they were manually traced. The scatter plots of Depth50 and Width50 are shown in Figure 6(a). For the average of each variety, Depth50 ranged from 5 cm to 23 cm and Width50 ranged from 3 to 14 cm. Broad-sense heritability of Depth50 was 0.77 and that of Width50 were 0.74. In previous studies, broad-sense heritability of root-related traits ranged from 0.14 to 0.54 in chickpea (*Cicer arietinum* L.) [47], from 0.50 to 0.92 in wheat (*Triticum aestivum* L.) [48], and from 0.66 to 0.89 in rice [49]. Compared to them, the broad-sense heritability of Depth50 and Width50 was relatively high. Overall, Depth50 and Width50 could be improved as a result of genetic modification. The Depth50 of IR64, Dro1-NIL, and KP were 8.1 cm, 11.2 cm, and 14.3 cm, respectively. This corroborates their different root distribution properties. We divided the 60 accessions into four groups: *japonica*, *indica*, *aus*, and admixed subspecies [30]. There were no significant differences between Depth50 and Width50 among subspecies (Figure 6(b)), raising the possibility that the root distribution was affected by local adaptation rather than subspecies divergence. Then, we divided the 60 accessions by clustering with Depth50 and Width50 (Figure S9). As a result, five clusters were obtained. Among them, cluster IV consisted of 13 accessions having higher Depth50, and cluster V consisted of 8 accessions having higher Width50. Although this analysis used one-year, one-location data, these results indicate that deep learning-based phenotypic analysis is applicable for evaluating root distribution parameters from field image data to find candidate materials with a characteristic root distribution.

## 4. Discussion

Root distribution in the field is one of the most important traits for crop production because it influences the efficiency of nutrient and water absorption and the consequent crop growth and yield [2, 5, 6]. However, phenotyping methods for root distribution are limited, and the procedures are laborious [10]. In recent years, CNN-assisted image analysis has been widely used for field research, for example, in seedling and panicle segmentation [50, 51], heading date estimation [27], and paddy field pest mapping [52]. The segmentation accuracy of CNN is high. For instance, it achieved over 90% accuracy for rice and weed segmentation [50] and the error in the heading date was only 0.8 days compared to manual observation [27]. Nevertheless, CNN-based field root research is not yet widely used. In this study, we simplified the estimation of root distribution from profile images by using a CNN. The overall root distribution (Figure 4) and root distribution parameters (Figure 5) were very similar between the manual and CNN protocols. Nonetheless, details of the predicted images differed from manually labeled images (Figure 3). We labeled using a pencil tool of 4-pixel width, regardless of the root width; it is possible that such a nonstrict manual tracing produced the root segmentation errors, reducing the Dice coefficient, but that the errors are local. Global parameters such as the root distribution were not affected. This means that the phenotypes involving percentage, and not only root distribution, could be quantified by a dataset with nonstrict manual tracing and CNN, which is an advantage for saving the time required for manual tracing.

There are many types of models for semantic segmentation, but the best model depends on the image type and tasks required. For example, SegNet [53] performed better than U-Net for rice and weed segmentation [50]; U-Net could not classify the rice and weeds properly compared to SegNet because U-net recognized rice and weeds simply as a green plant object. To the contrary, a previous study segmented chicory (*Cichorium intybus*) roots using U-Net [30], implying that U-Net is good at segmenting the roots in the soil. In this study, we also used U-Net to successfully segment rice root on trench profile images. As color variation of the roots and soil is limited, our CNN-based segmentation method is applicable not only for rice roots but also for the roots of other crops.

In the traditional trench profile method, root length density is measured directly in the field or in the laboratory after collecting soil blocks from the field [12, 22, 23]. To the contrary, CNN-based image analysis requires only image data, meaning that all we need to do outdoors is to take pictures after flushing the trench profile wall. During model construction, it took about 10 hours to manually make label data for 30 trench profile images; 10 images were used for model training, and 20 were used for model validation. Then, the trained model spent only 30 min to label the data of 204 trench images. This illustrates that CNN-based root segmentation is over 100 times faster than manual segmentation on the computational resources at our disposal; the program was run in a 64-bit Windows 7 computer (CPU: Intel® Xeon® CPU E3-1270 v5, GPU: NVIDIA Quadro M2000). Moreover, the possibility of human error during manual tracing is eliminated by using the CNN.

The trench profile method is a powerful method for root distribution surveys in the field because it is possible to simultaneously estimate vertical and horizontal root distribution. However, the standard technique of [10], making the trench and evaluating the root distribution parameters by hand, is so time-consuming that it is difficult to analyze large populations, which means that the date of acquisition differs greatly between the first and last samples. In this study, the last trench image was taken one month after the first trench image, but the root distribution of the reference variety, KP, was not influenced by acquisition date (Figure S8). Since the total root length of rice decreases after heading [54, 55] due to the arrest of crown root development [55], the best period for root distribution phenotyping is after the vegetative phase but before heading. It was considered that at the first acquisition date—late August in Japan—the rice was in the late vegetative stage or the reproductive stage in most accessions used in this study, suggesting that the root distribution did not change significantly due to differences in the acquisition date.

In this study, we applied a deep neural network to promote root distribution phenotyping. We calculated two root distribution parameters: Depth50 and Width50 (Figure 2). In general, it is considered that a higher Depth50 adapts to nutrients and water defect conditions and lower Depth50 and higher Width50 adapt to high input systems [5, 6]. Using these two parameters, we characterized root distribution of worldwide rice accessions and found a large variation (Figure 6). By integrating other field methods, such as the direct excavation method, with large monolith providing total root length and root diameter [42] and that with shovels providing rooting angle and root branching parameters [19, 20], more advanced root phenomics will be conducted.

## Supplementary Materials

Supplementary Materials Figure S1: field design in 2018. The gray squares indicate plots, and the numbers in the squares indicate the ID in Table S1. Each plot contained 20 hills, and the hill spacing was 1m × 1m. Figure S2: field design in 2019. The squares indicate plots, and the numbers in the squares indicate the ID in Table S1. Figure S3: a diagram of the neural network architecture used in this study. Figure S4: the diagram of model training. Figure S5: representative results of data augmentation. Figure S6: the datasets used for construction of the prediction model. Figure S7: representative results of the entire root segmentation of 8 rice accessions. Figure S8: influence of the acquisition date of trench profile images on the root distribution parameters. Figure S9: heatmap and dendrogram of Depth50 and Width50 among worldwide rice accessions from 2019. Table S1: the rice accessions used in this study. Table S2: data augmentation parameters.
