# Supplementary material for: A Deep Learning-Based Phenotypic Analysis of Rice Root Distribution from Field Images
Source: Plant Phenomics. 2020 Oct 16;2020:3194308. doi: 10.34133/2020/3194308 (PMC7706345; doi:10.34133/2020/3194308)
Supplement: Supplementary Materials — Figure S1: field design in 2018. The gray squares indicate plots, and the numbers in the squares indicate the ID in Table S1. Each plot contained 20 hills, and the hill spacing was 1m × 1m. Figure S2: field design in 2019. The squares indicate plots, and the numbers in the squares indicate the ID in Table S1. Figure S3: a diagram of the neural network architecture used in this study. Figure S4: the diagram of model training. Figure S5: representative results of data augmentation. Figure S6: the datasets used for construction of the prediction model. Figure S7: representative results of the entire root segmentation of 8 rice accessions. Figure S8: influence of the acquisition date of trench profile images on the root distribution parameters. Figure S9: heatmap and dendrogram of Depth50 and Width50 among worldwide rice accessions from 2019. Table S1: the rice accessions used in this study. Table S2: data augmentation parameters. [file 3194308.f1.docx]

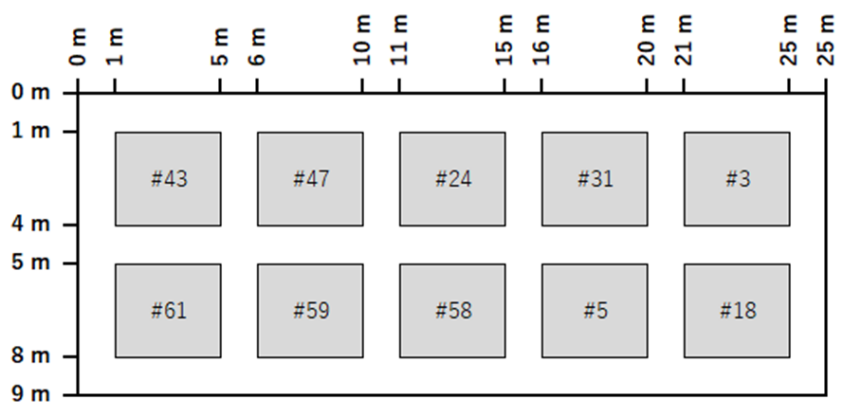


**Figure S1. Field design in 2018.** The gray squares indicate plots and the numbers in the squares indicate the ID in Table S1. Each plot contained 20 hills and the hill spacing was 1 m × 1 m.


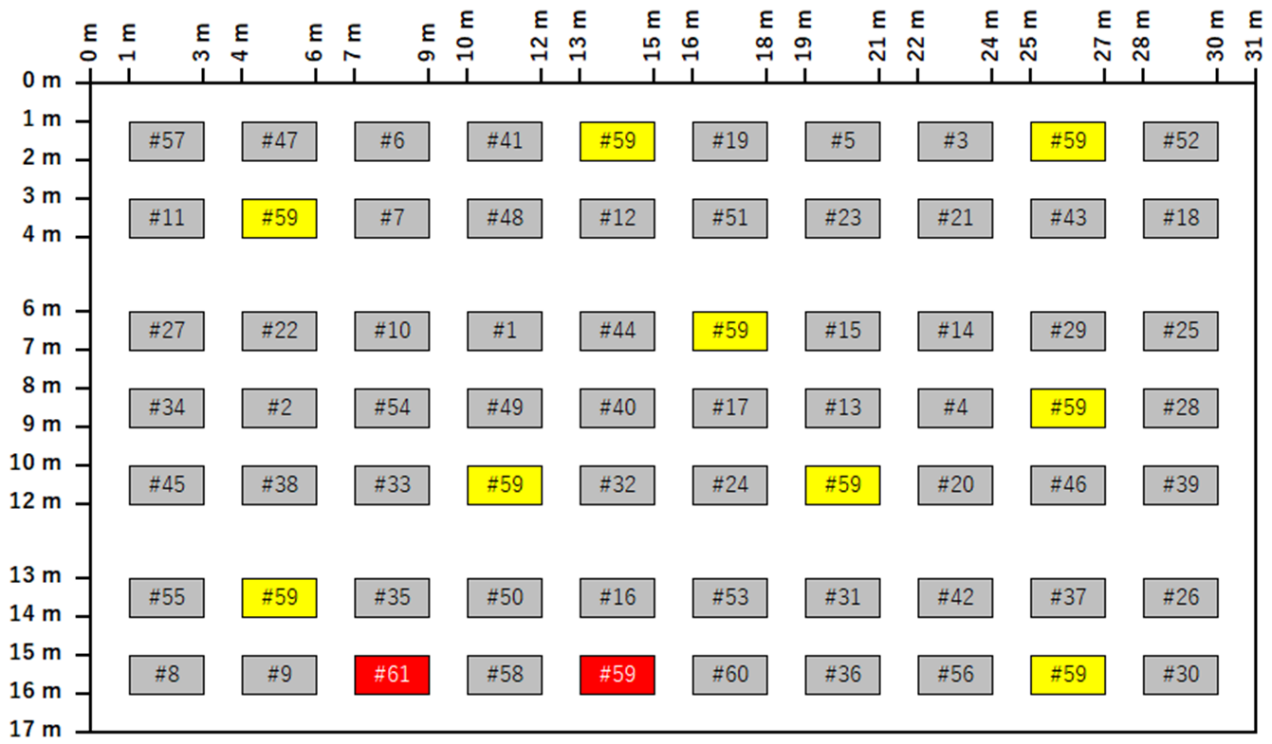


**Figure S2. Field design in 2019.** The squares indicate plots, and the numbers in the squares indicate the ID in Table S1. Each plot contained six hills, and the hill spacing was 1 m × 1 m. The yellow squares consisted of a single variety, Kinandang Patong (KP). The plots of the red squares were lost due to growth defects and soil collapse.


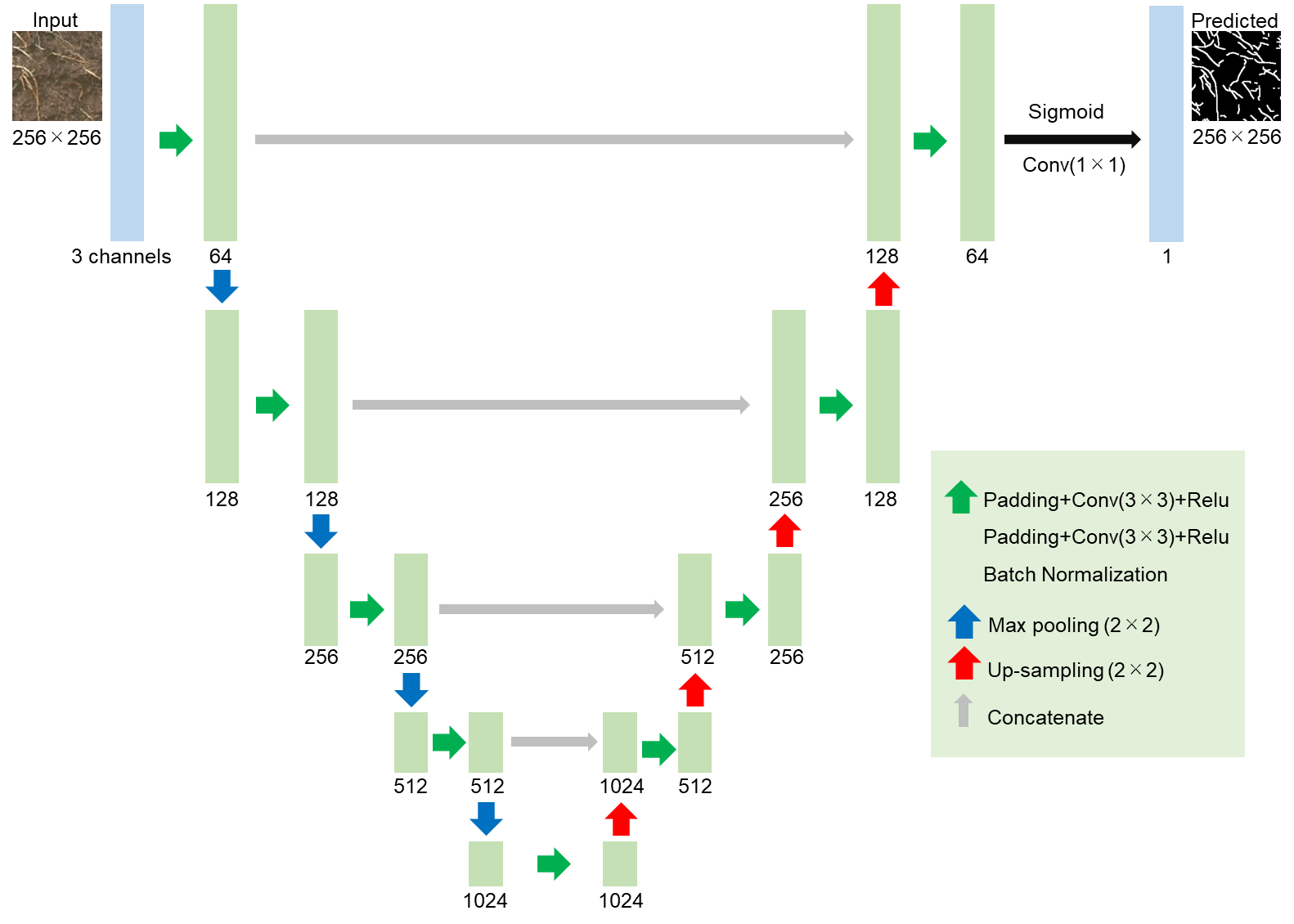


**Figure S3. A diagram of the neural network architecture used in this study.** The model was trained using the Adam optimizer, of which learning rate was 0.001, for 500 epochs (360 inputs per an epoch) using 360 training image sets from 2018.

**Figure S4. The diagram of model training.** Trench profile and labeled images were separated into 36 tiles, processed with data augmentation, and subjected to model training.


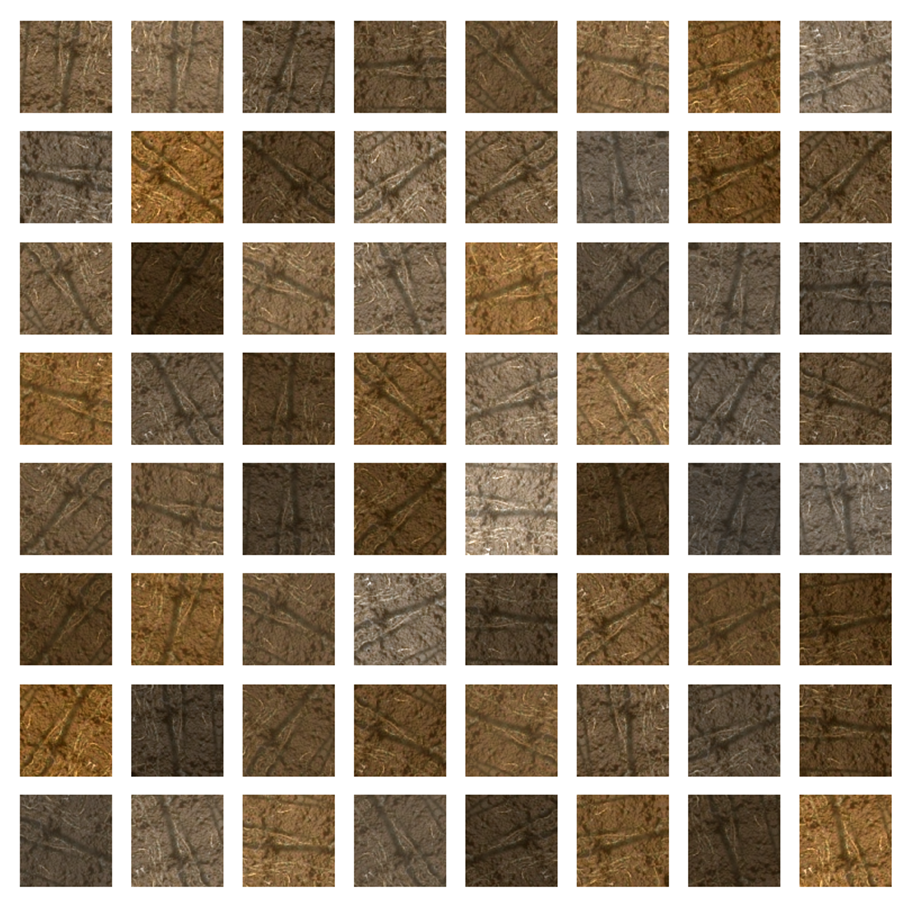


**Figure S5. Representative results of data augmentation.** A one-tile image was augmented into 64 tiles.


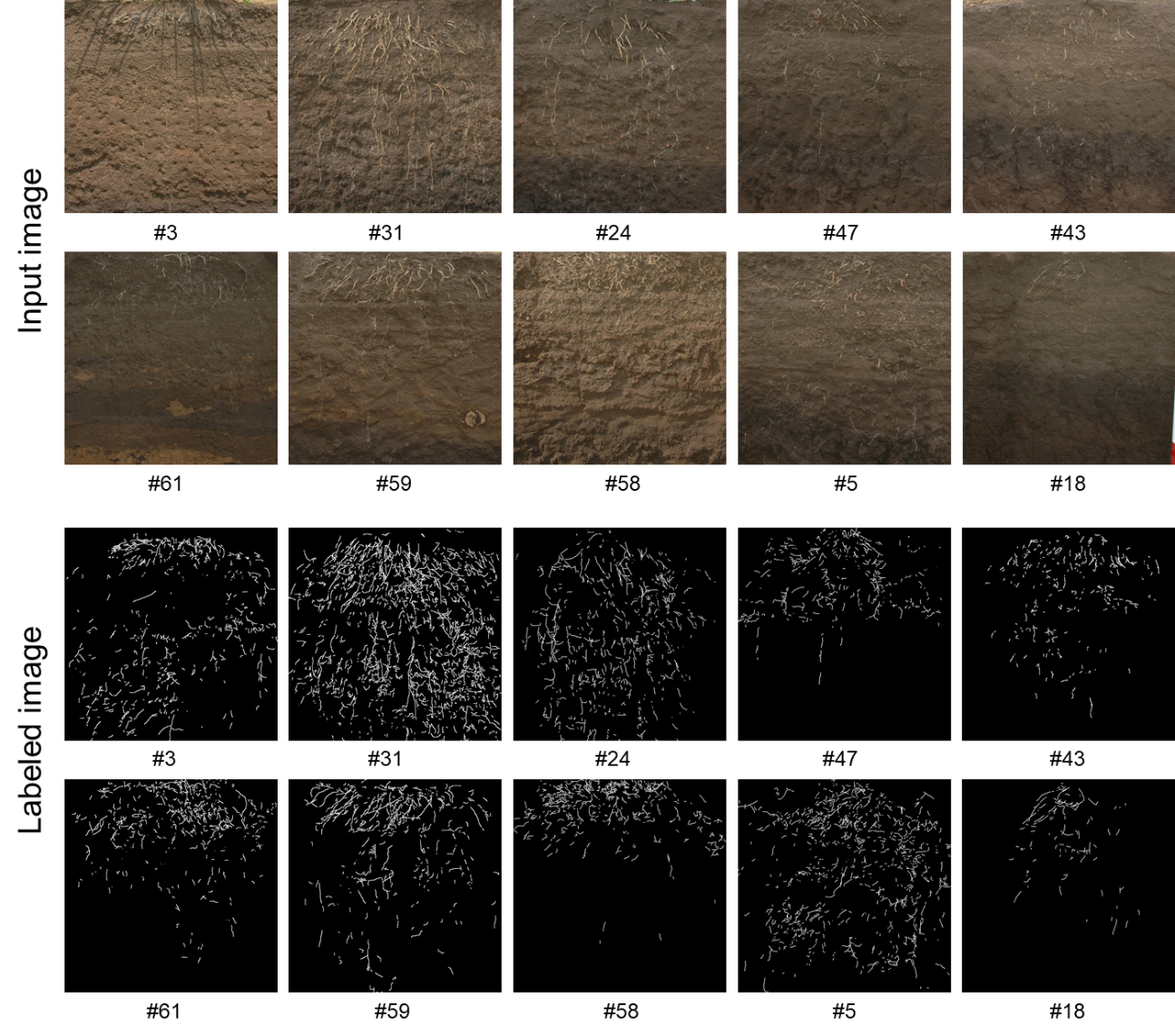


**Figure S6. The datasets used for construction of the prediction model.** The numbers shown at the bottom of the figures are accession ID.


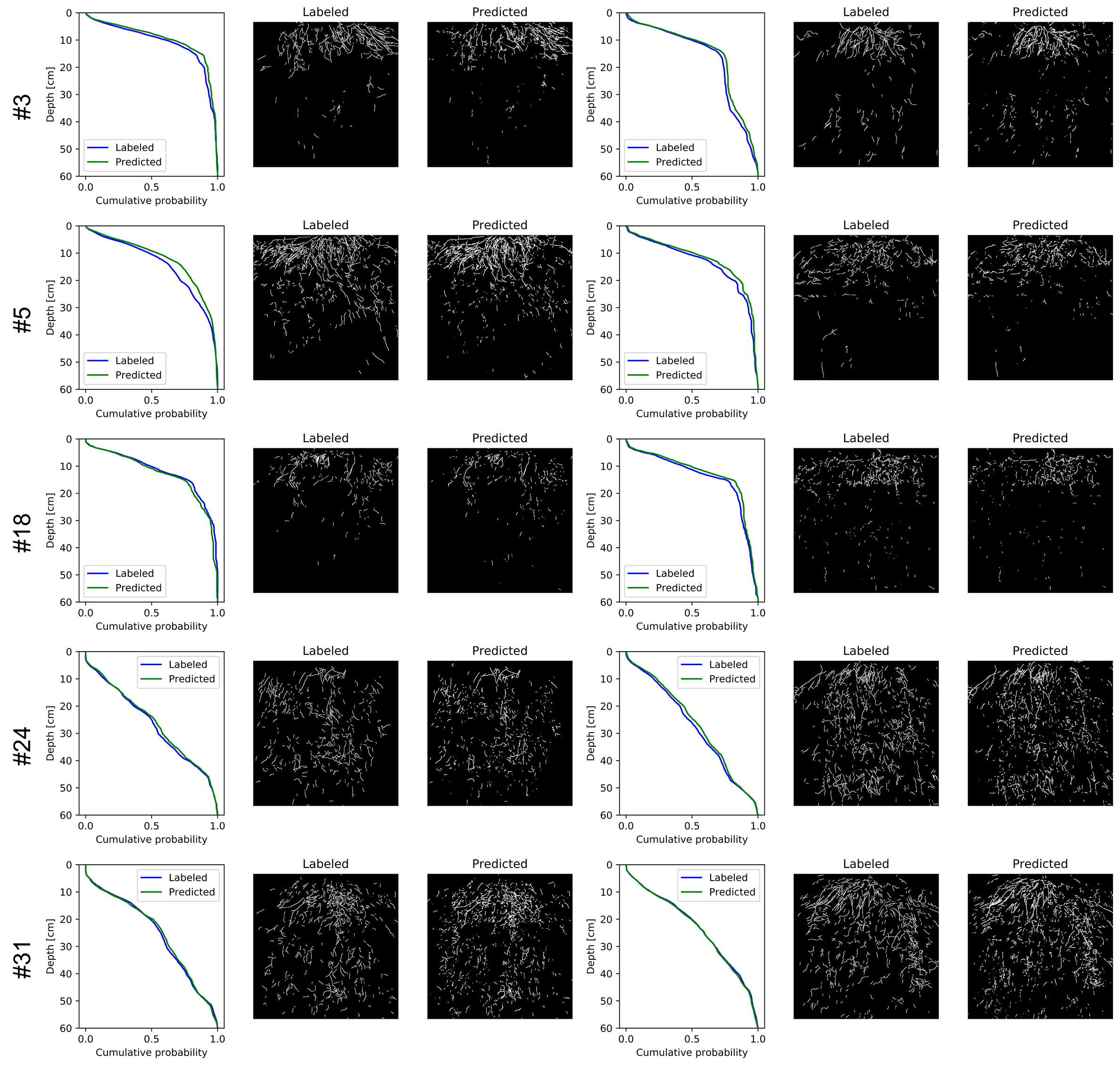


**Figure S7. Representative results of the entire root segmentation of 8 rice accessions.** The line graphs show the cumulative probability vs. depth. The lines of labeled and predicted images are included. Entire root segments that were manually labeled (Labeled) or labeled by trained model prediction (Predicted) are shown on the right. The numbers shown at the left denote accession ID.


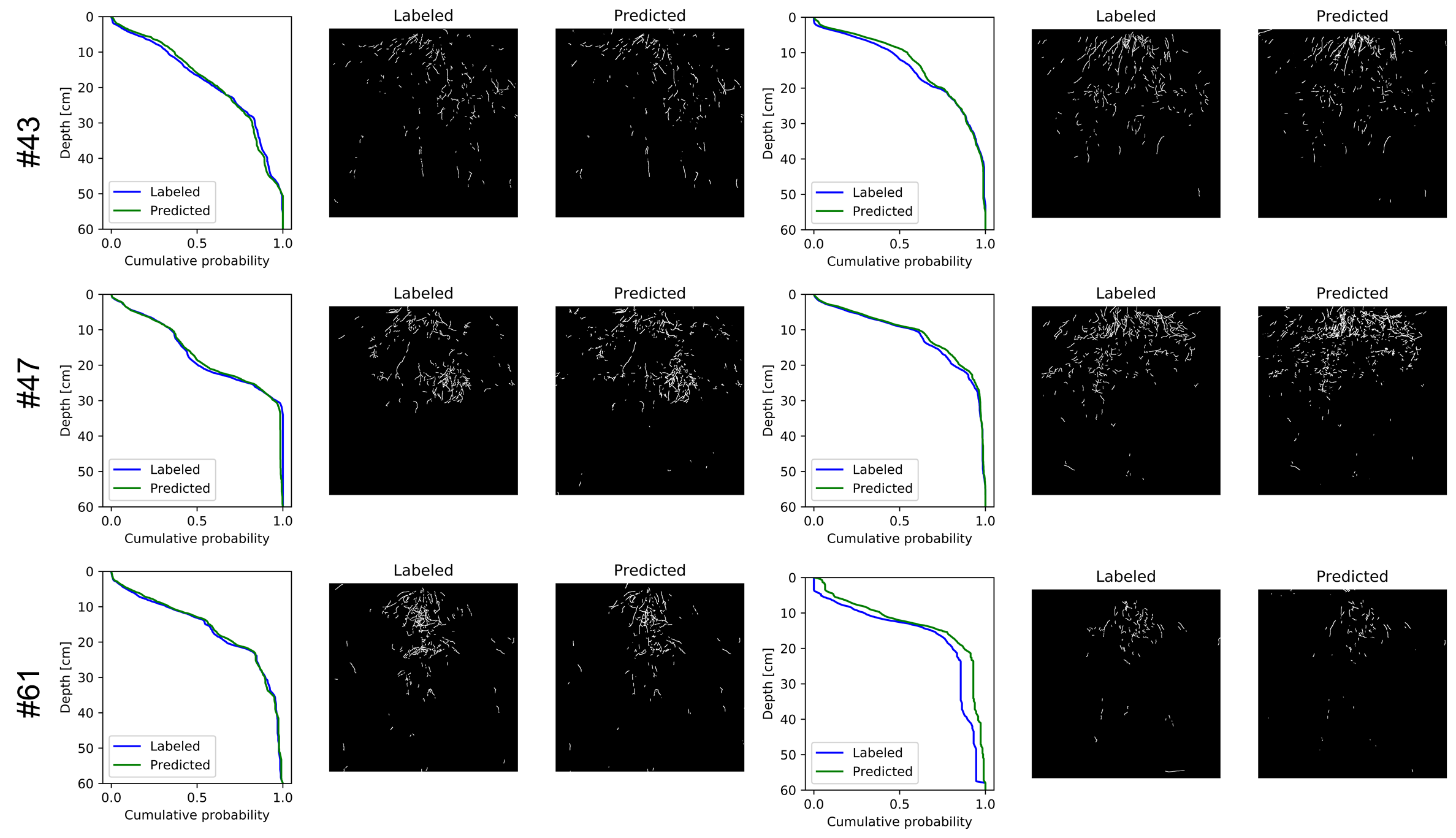


**Figure S7 (continued).**


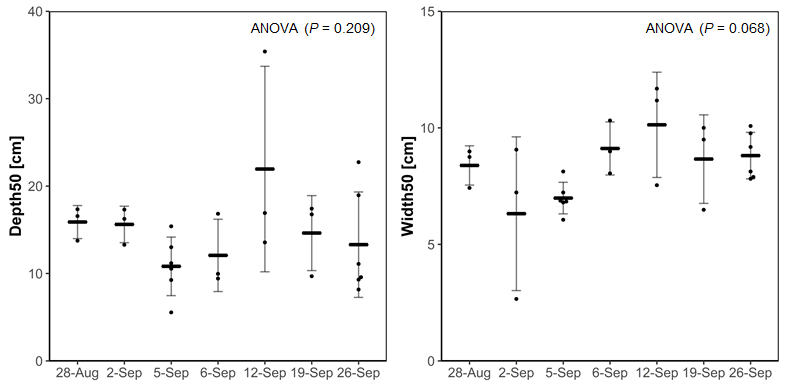


**Figure S8. Influence of the acquisition date of trench profile images on the root distribution parameters.** The center lines and the whiskers represent the mean and standard deviation, respectively. The constituents are marked as scatter points.


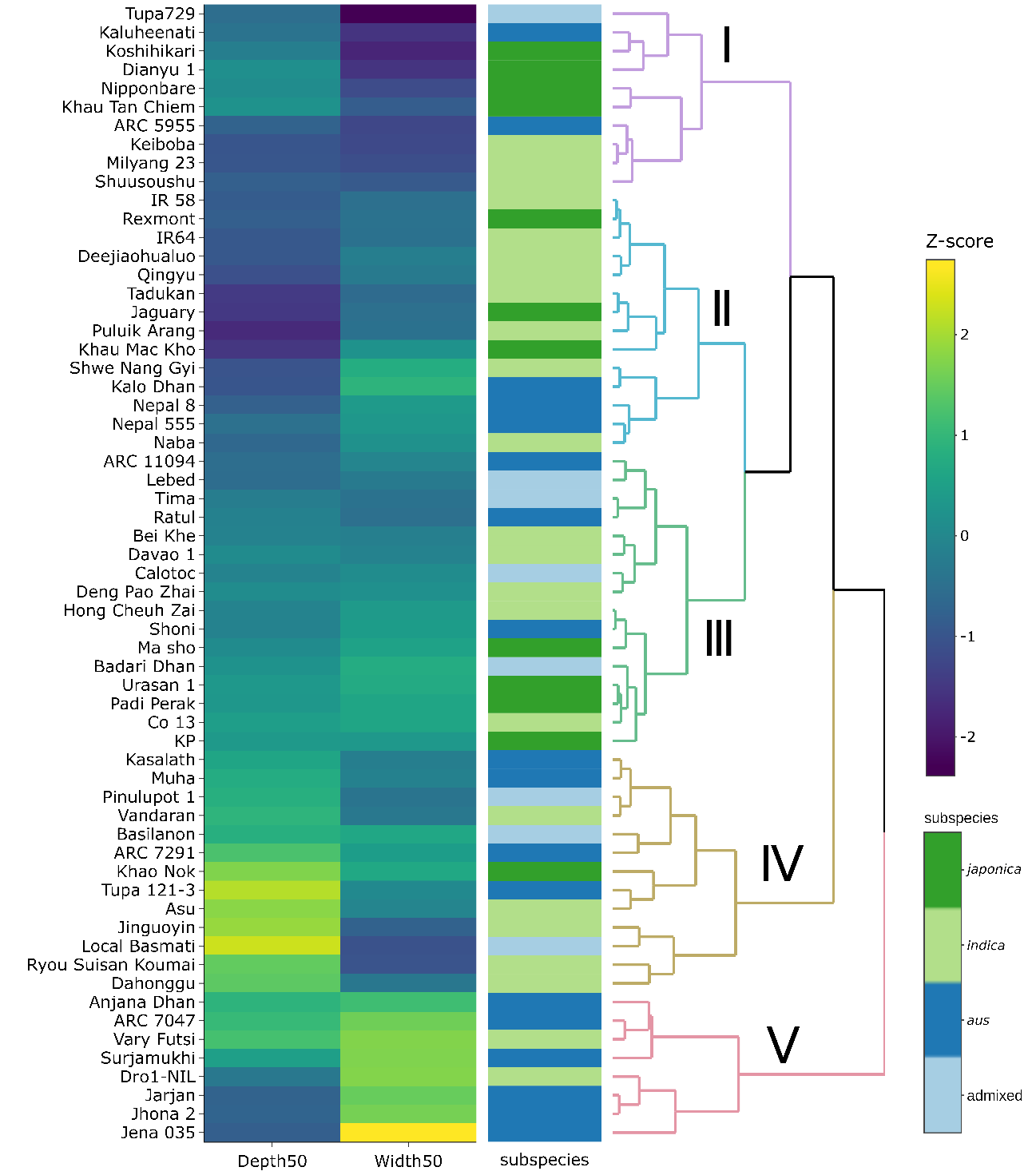
**Figure S9. Heatmap and dendrogram of Depth50 and Width50 among worldwide rice accessions from 2019.** Depth50 and Width50 were normalized with mean 0 and variance 1.

**Table S1. The rice accessions used in this study.**

| ID | Accession | Variety Name | Subspecies | 2018 | 2019 |
| --- | --- | --- | --- | --- | --- |
| 1 | WRC #1 | Nipponbare | temperate-*japonica* | No | Yes |
| 2 | WRC #2 | Kasalath | *aus* | No | Yes |
| 3 | WRC #3 | Bei Khe | *indica* | Yes | Yes |
| 4 | WRC #4 | Jena 035 | *aus* | No | Yes |
| 5 | WRC #5 | Naba | *indica* | Yes | Yes |
| 6 | WRC #6 | Puluik Arang | *indica* | No | Yes |
| 7 | WRC #7 | Davao 1 | *indica* | No | Yes |
| 8 | WRC #9 | Ryou Suisan Koumai | *indica* | No | Yes |
| 9 | WRC #0 | Shuusoushu | *indica* | No | Yes |
| 10 | WRC #11 | Jinguoyin | *indica* | No | Yes |
| 11 | WRC #12 | Dahonggu | *indica* | No | Yes |
| 12 | WRC #13 | Asu | *indica* | No | Yes |
| 13 | WRC #14 | IR 58 | *indica* | No | Yes |
| 14 | WRC #15 | Co 13 | *indica* | No | Yes |
| 15 | WRC #16 | Vary Futsi | *indica* | No | Yes |
| 16 | WRC #17 | Keiboba | *indica* | No | Yes |
| 17 | WRC #18 | Qingyu | *indica* | No | Yes |
| 18 | WRC #19 | Deng Pao Zhai | *indica* | Yes | Yes |
| 19 | WRC #20 | Tadukan | *indica* | No | Yes |
| 20 | WRC #21 | Shwe Nang Gyi | *indica* | No | Yes |
| 21 | WRC #22 | Calotoc | admixed | No | Yes |
| 22 | WRC #23 | Lebed | admixed | No | Yes |
| 23 | WRC #24 | Pinulupot 1 | admixed | No | Yes |
| 24 | WRC #25 | Muha | *aus* | Yes | Yes |
| 25 | WRC #26 | Jhona 2 | *aus* | No | Yes |
| 26 | WRC #27 | Nepal 8 | *aus* | No | Yes |
| 27 | WRC #28 | Jarjan | *aus* | No | Yes |
| 28 | WRC #29 | Kalo Dhan | *aus* | No | Yes |
| 29 | WRC #30 | Anjana Dhan | *aus* | No | Yes |
| 30 | WRC #31 | Shoni | *aus* | No | Yes |
| 31 | WRC #32 | Tupa 121-3 | *aus* | Yes | Yes |
| 32 | WRC #33 | Surjamukhi | *aus* | No | Yes |
| 33 | WRC #34 | ARC 7291 | *aus* | No | Yes |
| 34 | WRC #35 | ARC 5955 | *aus* | No | Yes |
| 35 | WRC #36 | Ratul | *aus* | No | Yes |
| 36 | WRC #37 | ARC 7047 | *aus* | No | Yes |
| 37 | WRC #38 | ARC 11094 | *aus* | No | Yes |
| 38 | WRC #39 | Badari Dhan | admixed | No | Yes |
| 39 | WRC #40 | Nepal 555 | *aus* | No | Yes |
| 40 | WRC #41 | Kaluheenati | *aus* | No | Yes |
| 41 | WRC #42 | Local Basmati | admixed | No | Yes |
| 42 | WRC #43 | Dianyu 1 | temperate-*japonica* | No | Yes |
| 43 | WRC #44 | Basilanon | admixed | Yes | Yes |
| 44 | WRC #45 | Ma sho | tropical-*japonica* | No | Yes |
| 45 | WRC #46 | Khao Nok | admixed-*japonica* | No | Yes |
| 46 | WRC #47 | Jaguary | admixed-*japonica* | No | Yes |
| 47 | WRC #48 | Khau Mac Kho | admixed-*japonica* | Yes | Yes |
| 48 | WRC #49 | Padi Perak | tropical-*japonica* | No | Yes |
| 49 | WRC #50 | Rexmont | tropical-*japonica* | No | Yes |
| 50 | WRC #51 | Urasan 1 | tropical-*japonica* | No | Yes |
| 51 | WRC #52 | Khau Tan Chiem | temperate-*japonica* | No | Yes |
| 52 | WRC #53 | Tima | admixed | No | Yes |
| 53 | WRC #55 | Tupa729 | admixed | No | Yes |
| 54 | WRC #57 | Milyang 23 | *indica* | No | Yes |
| 55 | WRC #98 | Deejiaohualuo | *indica* | No | Yes |
| 56 | WRC #99 | Hong Cheuh Zai | *indica* | No | Yes |
| 67 | WRC #100 | Vandaran | *indica* | No | Yes |
| 58 | IRGC #66970 | IR64 | *indica* | Yes | Yes |
| 59 | IRGC #23364 | Kinandang Patong | tropical-*japonica* | Yes | Yes |
| 60 | NA | Dro1-NIL | *indica* | No | Yes |
| 61 | NA | Koshihikari | temperate-*japonica* | Yes | No |

**Table S2. Data augmentation parameters.**

| Action | Description | Input image | Labeled image |
| --- | --- | --- | --- |
| rotation | Rotates the image from 0 to 90 degree. | Yes | Yes |
| width shift | Shifts the image horizontally by from 0 to 5% | Yes | Yes |
| height shift | Shifts the image vertically by from 0 to 5% | Yes | Yes |
| shear | Performs shear mapping by from 0 to 5% | Yes | Yes |
| zoom | Enlarges the image by from 0 to 5% | Yes | Yes |
| horizontal flip | Flips the image horizontally | Yes | Yes |
| vertical flip | Flips the image vertically | Yes | Yes |
| intensity | Of the window width, adjusts the minimum from 0 to 31 and the maximum from 160 to 255 | Yes | No |
| gamma index | Performs gamma correction by from -5 to 5% | Yes | No |
| chroma | Adjusts chroma of the image by -20 to 20% | Yes | No |
